# ONC201 exerts oncogenic effects beyond its mitochondria-disturbing role in neuroblastoma subsets

**DOI:** 10.1007/s00109-025-02541-0

**Published:** 2025-04-10

**Authors:** Jyun-Hong Jiang, Yu-Han Lin, Pei-Lin Liao, Ting-Ya Chen, Hui-Ching Chuang, Chao-Cheng Huang, Wen-Ming Hsu, Jiin-Haur Chuang, Wei-Shiung Lian

**Affiliations:** 1 Department of Pediatric Surgery, Kaohsiung Chang Gung Memorial Hospital, Graduate Institute of Clinical Medical Sciences, College of Medicine, Chang Gung University, Kaohsiung, Taiwan; 2 Center for Mitochondrial Research and Medicine, College of Medicine Chang Gung University, Kaohsiung Chang Gung Memorial Hospital, Kaohsiung, Taiwan; 3 Department of Otolaryngology, Kaohsiung Chang Gung Memorial Hospital, Chang Gung University College of Medicine, Kaohsiung, Taiwan; 4 Department of Pathology, Kaohsiung Chang Gung Memorial Hospital, College of Medicine, Chang Gung University, Kaohsiung, Taiwan; 5 Department of Surgery, National Taiwan University Hospital, National Taiwan University College of Medicine, Taipei, Taiwan; 6 Core Laboratory for Phenomics & Diagnostics, Department of Medical Research, Kaohsiung Chang Gung Memorial Hospital, Chang Gung University, Kaohsiung, Taiwan

**Keywords:** Neuroblastoma, ONC201, Non-*MYCN*-amplified cell line, LGR5, ATRX

## Abstract

Neuroblastoma (NB) is a formidable challenge in pediatric oncology due to its intricate molecular landscape, necessitating multifaceted therapeutic approaches. ONC201 is an imipridone antibiotic compound with a promising drug candidate leveraging its potent anticancer properties against the mitochondrial proteases ClpP and ClpX. Despite demonstrating early clinical promise, particularly in *MYCN*-amplified NB, its efficacy in non-*MYCN*-amplified NB remains a subject worthy of investigation. In this study, we extended the coverage of ONC201 to treat non-*MYCN*-amplified NB, and our data implicated ONC201’s inability to reduce tumor growth in animal models harboring SK-N-AS or SK-N-FI cell lines. Interestingly, ONC201 induced the expression of oncogenic markers c-Myc and LGR5 while downregulating the tumor suppressor ATRX. While it fails to attenuate tumor neovascularization in non-*MYCN*-amplified NB xenografts, its effectiveness differs from that of its MYCN-amplified counterpart. Rho zero (ρ0)-SK-N-AS cells treated with ONC201 showed comparable observed trends in parental SK-N-AS cells, including LGR5 upregulation and ATRX downregulation, suggesting that ONC201’s multifaceted actions extend beyond mitochondrial targets. Our elucidation highlights the need to discern molecular signatures when deploying ONC201 monotherapy against NB, which lacks *MYCN-*amplification.

**Supplementary Information:**

The online version contains supplementary material available at 10.1007/s00109-025-02541-0.

## Key messages

• ONC201 does not suppress growth in non-*MYCN*-amplified NB xenografts.

• ONC201 induces oncogenes c-myc and LGR5 and lowers ATRX.

• ONC201’s impact extends beyond mitochondrial targeting.

**Supplementary Information:**

The online version contains supplementary material available at 10.1007/s00109-025-02541-0.

## Introduction

Neuroblastoma (NB) is a genetically diverse pediatric tumor arising from neural crest-derived cells. It is characterized by pleomorphic cells and differentiation defects caused by genomic and epigenetic damage, associated with a wide range of clinical outcomes, from localized or self-resolving to extensively metastatic diseases. Genetic alterations in NB have prevented the development of targeted therapies, requiring combination therapy or intense multimodal treatment in patients with high-risk disease [1]. Although the combination of anti-disialoganglioside (anti-GD2) antibody plus isotretinoin showed promising results in patients with high-risk NB, the treatment was associated with substantial pain, requiring management with high-dose opioid infusion [2]. Thus, it is important to develop novel therapies or new combination treatments for high-risk NB with reduced side effects.

ONC201 is a dopamine receptor D2 (DRD2) antagonist and caseinolytic mitochondrial matrix peptidase proteolytic subunit P (ClpP) agonist that induces cancer cell apoptosis, cell cycle arrest, and antiproliferative effects, including effects on solid tumors and hematological malignancies [3]. Over 30 Phase I and Phase III clinical trials involving ONC201, either alone or in combination with other treatments, are currently active or have been completed (https://clinicaltrials.gov/). In phase II studies, ONC201 induced durable tumor regression in adults with recurrent H3 K27M-mutant glioma and in neuroendocrine tumors, including pheochromocytoma-paraganglioma and desmoplastic small round cell tumors [3, 4].

In our previous study [5], ONC201 suppressed the expression of ClpP and caseinolytic mitochondrial matrix peptidase proteolytic subunit X (ClpX), while downregulating the mitochondrial respiratory chain subunits succinate dehydrogenase complex iron sulfur subunit B (SDHB) and NADH/ubiquinone oxidoreductase core subunit S1 (NDUFS1); these effects resulted in energy depletion and increased reactive oxygen species (ROS) levels, along with suppression of NB cell growth, regardless of *MYCN* amplification status. However, ONC201 only inhibited tumor development in xenograft tumors derived from NB cells with amplified *MYCN*; this inhibition correlated with a substantial reduction in MYCN protein levels and the reactivation of ATRX expression. In vivo application of ONC201 failed to produce a treatment effect for non-*MYCN*-amplified NB xenografts similar to the effect observed for *MYCN*-amplified NB xenografts; the contributing factors are unknown.

ONC201 monotherapy failed to produce objective responses in a phase II study of recurrent or refractory metastatic breast or endometrial cancer [6]. The survival of D425 cells, medulloblastoma cells characterized by high c-Myc expression, was unaffected by ONC201 treatment [7]. This finding suggested that c-Myc oncogene expression reduces the effect of ONC201 treatment. Some other factors are obviously important for maximal ONC201 efficacy in the treatment of NB. Preclinical trials combining ONC201 with the glycolytic inhibitor 2-deoxyglucose (2DG) have shown dual metabolic reprogramming and synergistic effects that inhibit both the proliferation and migration of glioblastoma cells [8]. Previous research has indicated that NB cells with amplified *MYCN* are highly glycolytic, implying that 2DG can inhibit the growth of NB cells [9]. 2DG might also concurrently affect both cancer cells and endothelial cells in NB xenografts in mice, independent of the *MYCN* amplification status [10].

In this study, we demonstrated the pluripotency of ONC201 in the treatment of subgroups of NB that were free of the hurdle of disturbing mitochondria and thus could not collaborate with 2DG to potentiate their effects on the growth of NB xenografts.

## Materials and methods

### Cell culture and relative reagents

Different types of human NB cell lines, such as SK-N-AS, SK-N-FI, and SK-N-DZ were performed for the study and obtained from the American Type Culture Collection (Manassas, VA, USA). Cell lines were routinely grown in culture medium (DMEM, Dulbecco’s Modified Eagle’s Medium) with 10% FBS (heat-inactivated fetal bovine serum), GlutaMAX™, nonessential amino acids, and an antibiotic–antimycotic (Thermo Fisher Scientific, Waltham, MA, USA). The cells were maintained at 37 °C in the incubator with 5% CO_2_ humidified atmosphere. ONC201 was purchased from Sigma-Aldrich (St. Louis, MO, USA). Antibodies for ATRX were procured from Abcam (Cambridge, MA, USA), β-actin from Santa Cruz Biotechnology (Santa Cruz, CA, USA), LGR5 from GeneTex (Taipei, Taiwan), c-myc, and Ki- 67 from Cell Signaling Technology (Danvers, MA, USA).

### NB xenografted animal model

Male NOD/SCID mice, aged 4 weeks, were procured from the National Laboratory Animal Center (Taipei, Taiwan). The experimental use of animals was cleared by the Institutional Animal Care and Use Committee of Kaohsiung Chang Gung Memorial Hospital, Taiwan (approval number: 2019091805). To establish human NB xenografts in the mice, SK-N-AS cells (1 × 10^6^), SK-N-FI cells (1 × 10^7^), or SK-N-DZ cells (1 × 10^7^) were individually injected subcutaneously into the right flank of each mouse. After 7 days of implantation, the mice were randomly assigned to different treatment groups. They received either a control treatment (blank culture medium), ONC201 (50 µg/g), 2DG (50 µg/g), or a combined treatment of ONC201 and 2DG (50 µg/g each) administered via intraperitoneal injection once a week for 4 weeks, following which they were euthanized. Tumor size and weight were recorded as previously described [11]. Tissue samples were fixed by formalin overnight and embedded in paraffin for subsequent immunohistochemical staining.

### Terminal deoxynucleotidyl transferase dUTP nick end-labeling assay

The impact of ONC201 on NB cell viability was quantified using the TUNEL kit (In Situ Cell Death Detection Kit by Roche, Germany) detection. In brief, cells were fixed with 4% paraformaldehyde for 10 min and then incubated with a terminal deoxynucleotidyl transferase and fluorescein-dUTP at 37 °C and subsequently counterstained with DAPI (Southern Biotech, Birmingham, USA) for fluorescence microscopy analysis. The apoptotic index was calculated as the ratio of TUNEL-positive cells to DAPI-positive cells in three randomly selected microscopic fields.

### Western blotting analysis

Post-treatment with 5 µM ONC201 for 48 h, proteins were extracted from NB cells for analysis. The proteins were separated by SDS-PAGE and transferred to polyvinylidene difluoride (PVDF) membranes. These membranes were blocked with 5% milk in tris-buffered saline with Tween 20 (TBS-T) for 1 h, then incubated with primary antibodies and HRP-conjugated secondary antibodies for 1 h each. Protein complexes were detected by an enhanced chemiluminescence kit (Amersham Pharmacia Biotech, Sweden) and recorded on X-ray film.

### Immunohistochemical analysis and scoring

ATRX in NB xenograft tissues and human NB samples were analyzed. Tissue sections were deparaffinized and blocked with 3% hydrogen peroxide, and antigen retrieval was performed using 0.01 M citrate buffer in the microwave at approximately 95 °C/10 min. After washing with PBS, sections were incubated with primary antibodies, followed by a peroxidase-conjugated polymer kit (Zymed Laboratories, San Francisco, CA) reaction for 30 min. Diaminobenzidine (DAB; Sigma, St. Louis, MO) was used for color development. Sections were counterstained with Gill’s hematoxylin, dehydrated, and mounted. Staining intensity and proportion were scored from 0 (none) to 3 (strong) and 0 (no positive cells) to 4 (81–100% positive cells), respectively. Statistically, these scores are multiplied and summed to obtain a final immunoreactivity score ranging from 0 to 12.

### Generation and characterization of ρ^0^ cells from NB SK-N-AS

The SK-N-AS NB cells were cultured in media containing 50 ng/ml ethidium bromide (EtBr) to remove mitochondrial DNA (mtDNA) for 3 months and isolate single clones through continuous limiting dilution. The NB ρ0 cells lacking mtDNA were characterized based on mtDNA copy number, expression of mtDNA-encoded proteins, and cellular oxygen consumption.

### Human NB tissue biopsies

Tissue samples from 26 patients diagnosed with neuroblastoma, ganglioneuroblastoma, and ganglioneuroma between 2005 and 2022 were obtained from the pathology archives of Chang Gung Memorial Hospital in Kaohsiung, Taiwan. All patients were under 19 years of age at diagnosis. The classification of these cases was based on the International Neuroblastoma Staging System [12], and the histological classification followed the guidelines of the International Neuroblastoma Pathology Committee [13]. The classification system used evaluates the proportion and maturity of NB cells and the presence of ganglion cells, classifying tumors into five types: undifferentiated neuroblastoma, poorly differentiated neuroblastoma, differentiated neuroblastoma, ganglioneuroma, and ganglioneuroma.

### Quantification of endothelial cell density after isolectin B4(IB4) staining

Endothelial cell density analysis in the xenografts tissue section was measured by staining with isolectin IB4-biotin conjugates (Molecular Probes). In brief, tissue slides were dewaxed, antigen retrieval was conducted, immunoreactive with antibodies in the sections, and the reaction color was measured using a DAB detection system kit. Five images (× 200 magnification) were selected from each tumor section to determine endothelial cell density, and the average positive staining area per total pixel was calculated.

### Trypan blue exclusion assay

For ONC201 treatments, after 24–96 h, cells were stained with 0.4% Trypan Blue (Gibco, Thermo Fisher Scientific Inc.) for 5 min and analyzed by a Zeiss microscopic (AxioVision, USA); for counted viable cells, which exclude Trypan Blue, they appeared clear in the cytoplasm, whereas non-viable cells absorbed the dye and displayed a blue cytoplasm. Counts of viable and non-viable cells were separately recorded to calculate the proportion of cell death.

### Flow cytometry for detection of apoptosis

To evaluate cellular apoptosis, a staining procedure was performed using fluorescein isothiocyanate (FITC)-conjugated annexin V and propidium iodide (PI) as per the manufacturer’s instructions (BD Pharmingen, BD Biosciences, San Jose, CA, USA). Post-treatment, the cells were suspended in an annexin-V-binding buffer and stained with annexin V/PI for 15 min. A FACS Calibur flow cytometer (BD Biosciences) was used for data collection. Cells positive for annexin V and either negative or positive for PI were identified as undergoing apoptosis.

### Statistical analysis

Statistical analyses were conducted using two-tailed Student’s *t*-tests to demonstrate mean differences between groups. Multiple comparisons were assessed with one-way or two-way ANOVA followed by nonparametric post-hoc Tukey’s *t*-tests. Data are presented as mean ± standard deviation (SD) or mean ± standard error of the mean (SEM), with significance defined as *P* values < 0.05 or 0.001. These findings represent a minimum of three independent experiments.

## Results

### ONC201 treatment failed to inhibit development of non-MYCN-amplified NB xenograft tumors

**Fig. 1:**
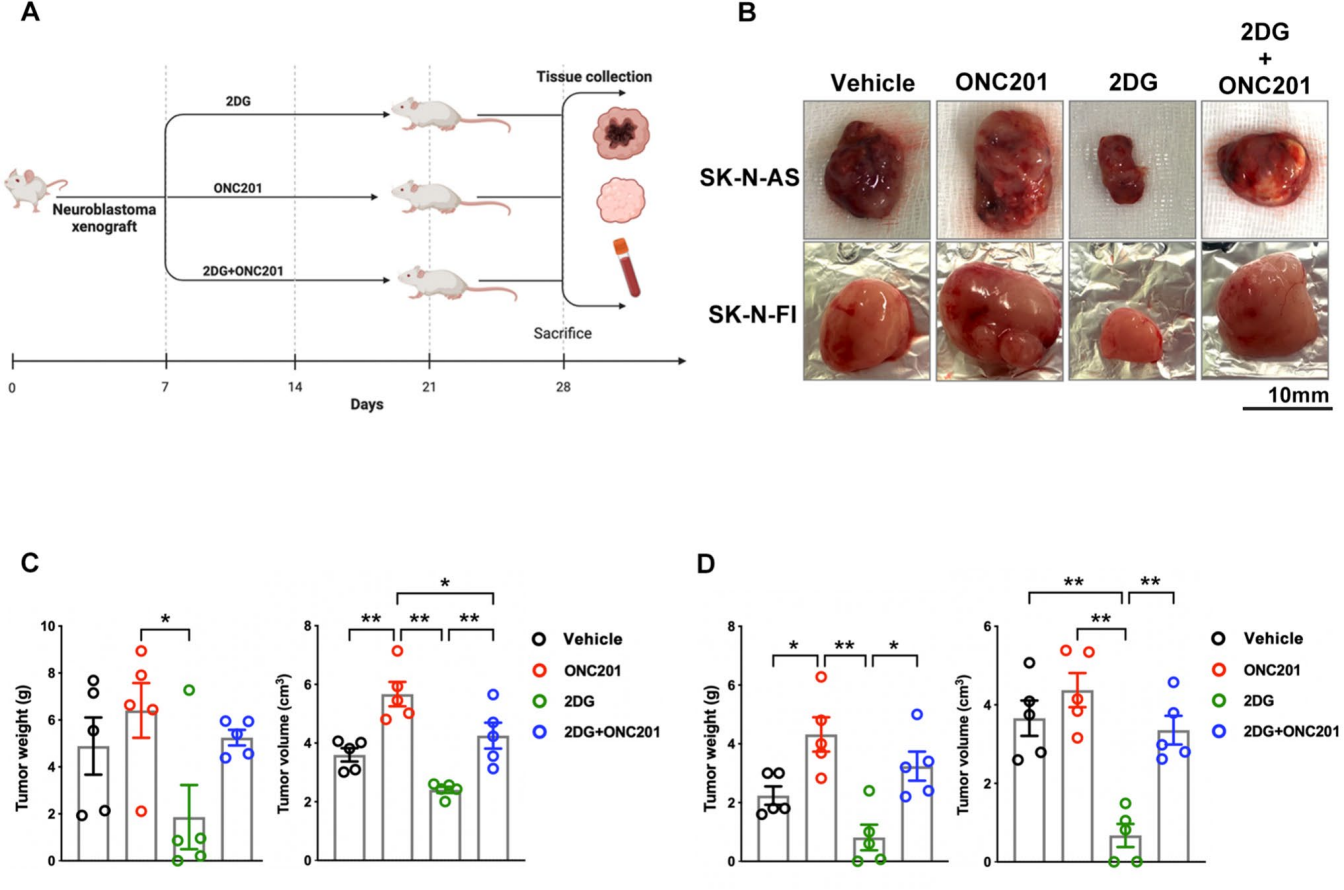
Effects of ONC201 and 2DG on NB xenografts in NOD/SCID mice. **A** Experimental schematic showing the experimental design, treatment schedules, and analysis process. **B** Measurement of tumor occurrence after subcutaneous injection of NB cells with or without ONC201 and/or 2DG treatment. **C** Quantification of tumor weight and volume in SK-N-AS cell group. **D** Quantification of tumor weight and volume in SK-N-FI cell group. Dots represent the numbers in each group, and colors indicate different groups. Tumor growth and volume quantifications are represented as mean ± SEM and are considered significant at *P < 0.05 and **P < 0.01, calculated using ordinary one-way ANOVA and Tukey’s multiple comparison test

To determine whether ONC201 modulates NB development, we evaluated the therapeutic efficacies of ONC201 and 2DG in non-*MYCN*-amplified xenograft tumors. NOD/SCID mice were injected with non-*MYCN*-amplified SK-N-AS or SK-N-FI cells or *MYCN*-amplified SK-N-DZ cells and treated with ONC201, 2DG, or a combination of both every 7 days for 4 weeks (Fig. 1A). Administration of 2DG (50 µg/g) significantly reduced tumor weight and volume in the non-*MYCN*-amplified SK-N-AS and SK-N-FI groups, whereas treatment with ONC201 (50 µg/g) resulted in a significant increase in tumors and counteracted the inhibitory effect of 2DG on NB development, as illustrated in the tumor image (Fig. 1B) along with the quantification of weight and volume (Fig. 1C and D). Although the potent antitumor activity of ONC201 was demonstrated in vitro [14] and in vivo studies [5], its significant therapeutic efficacy in the heterogeneous pathology of NB still needs to be further investigated.

### ONC201 exerted an oncogenesis effect in non-MYCN-amplified NB xenograft

**Fig. 2:**
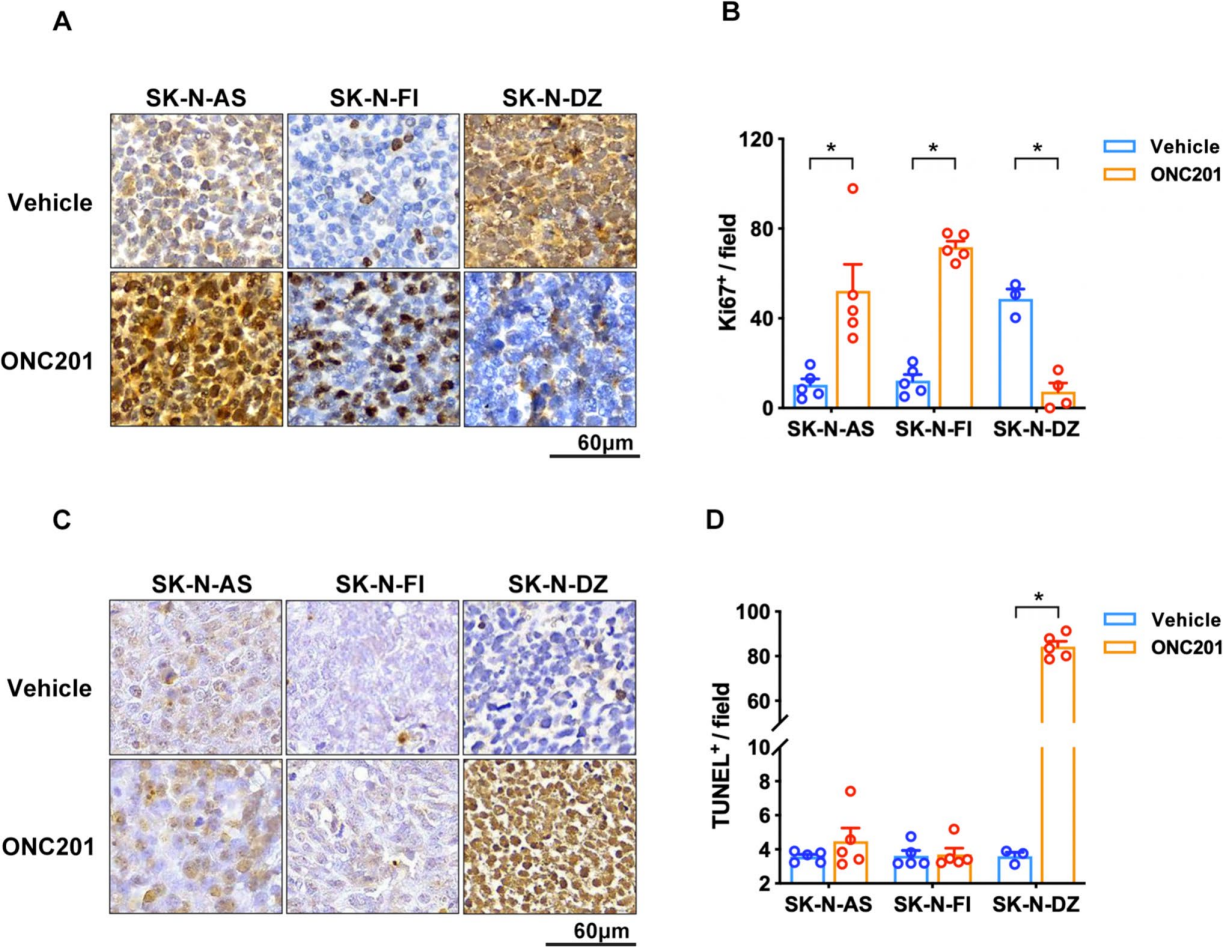
ONC201 induced tumorigenesis. **A** Immunohistochemical staining to examine the expression of Ki- 67 and **C** TUNEL staining in tissue sections. **B** Ki- 67 expression was significantly upregulated by ONC201, whereas **D** apoptosis was unaffected. Dots represent the numbers in each group, and colors indicate different groups. Data are presented as means ± SEM, and differences are considered significant at *P < 0.05. A two-group comparison was conducted utilizing an unpaired two-tailed *t*-test

To evaluate the impact of ONC201 on tumor development in NB xenografts, we examined expression of the cellular proliferation marker Ki- 67 in xenograft tissue sections. The results of IHC staining and quantification showed significantly increased Ki- 67 expression in ONC201-treated non-*MYCN*-amplified SK-N-AS and SK-N-FI xenograft groups than in the *MYCN*-amplified SK-N-DZ group (Fig. 2A and B). We examined apoptosis of xenograft tissue after ONC201 treatment. The results of TUNEL analysis showed no changes in the ratios of apoptotic cells in SK-N-AS and SK-N-FI xenograft groups with or without ONC201 treatment (Fig. 2C and D), whereas the level of apoptosis was significantly increased in the SK-N-DZ group with ONC201 treatment. These results indicated that ONC201 treatment does not prevent non-*MYCN*-amplified NB xenograft growth.

### ONC201 promoted c-Myc and LGR5 protein expression in non-MYCN-amplified NB cell lines and xenograft tissues

**Fig. 3:**
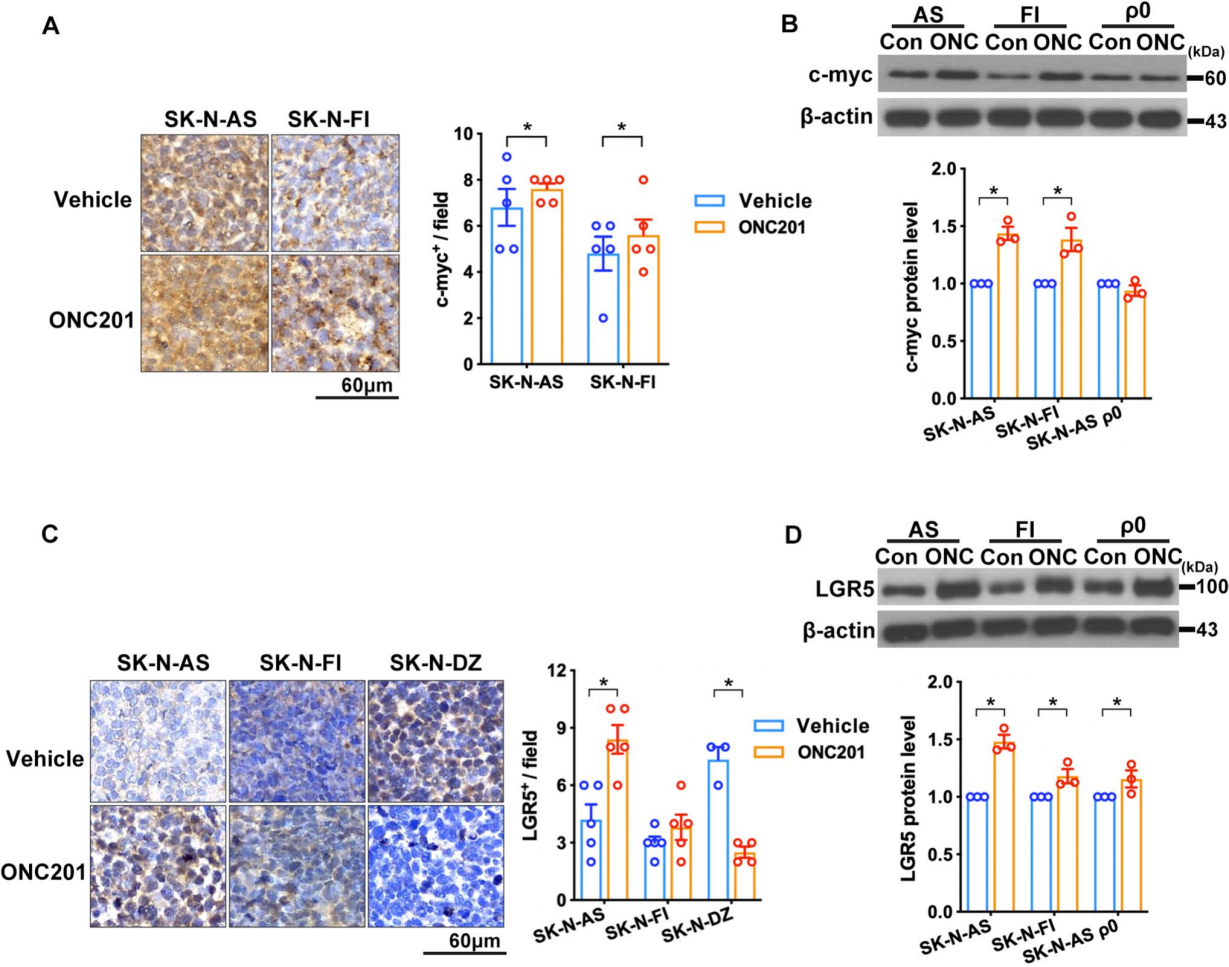
ONC201 increased c-Myc and LGR5 expression in non-*MYCN*-amplified cells*.* **A** c-Myc expression was increased in ONC201-treated (ONC) SK-N-AS and SK-N-FI xenograft tissues. **B** Immunoblots showing c-Myc expression in SK-N-AS and SK-N-FI cells after treatment with ONC201. **C** LGR5 expression was increased in ONC201-treated SK-N-AS xenograft tissue. **D** Immunoblots showing LGR5 expression in SK-N-AS, SK-N-FI, and SK-N-AS ρ^0^ cells after treatment with ONC201. Dots represent the numbers in each group, and colors indicate different groups. Data are presented as means ± SEM, and differences are considered significant at *P < 0.05. A two-group comparison was conducted utilizing an unpaired two-tailed *t*-test

The disruption of *MYCN* plays a crucial role in the development of NB and is strongly associated with poor prognosis [15]. The view of transcriptome in NB cells shows enrichment for canonical *MYC* target gene transcripts, which promote functional protein synthesis and control processes such as cell growth, cell cycle progression, mitochondrial bioenergetic synthesis, and metabolism [16, 17]. Therefore, we evaluated the expression of c-Myc in NB xenografts treated with ONC201. Immunohistochemistry analysis revealed high c-Myc expression in NB xenograft tissue in the groups treated with ONC201 (Fig. 3A). Additionally, ONC201 significantly increased c-Myc expression in SK-N-AS and SK-N-FI cells but not in mtDNA-depleted SK-N-AS cells (ρ^0^ cells) (Fig. 3B). Furthermore, LGR5 is a marker of stem cell proliferation and cellular behavior (e.g., self-renewal, invasion, and migration); it is positively correlated with β-catenin, cyclin D1, and c-Myc expression [18]. To investigate whether LGR5 is involved in the development of NB, we evaluated its expression under relevant experimental conditions. IHC analysis showed that LGR5 expression was significantly increased in the SK-N-AS group treated with ONC201, whereas its expression was reduced in the SK-N-DZ group (Fig. 3C). Western blotting analysis showed that the level of LGR5 expression was significantly increased in SK-N-AS, SK-N-FI, and ρ^0^ cell groups treated with ONC201 (Fig. 3D). ONC201 promoted c-Myc and LGR5 protein expression in non-*MYCN*-amplified NB cells. These findings are relevant for the development of NB in animal xenograft models.

### ONC201 promoted ATRX protein expression in non-MYCN-amplified NB cell lines and xenograft tissues

**Fig. 4:**
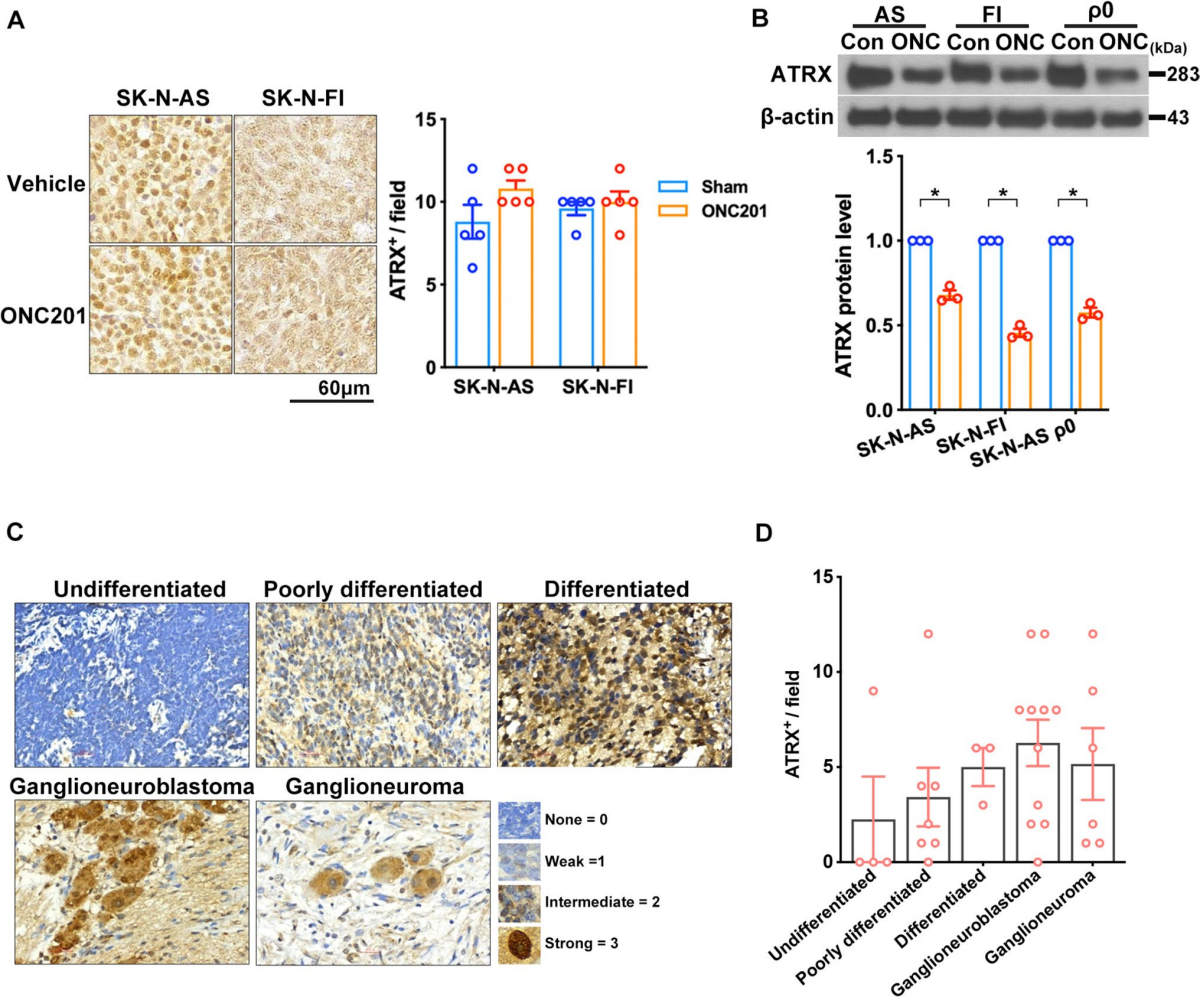
Analysis of ATRX expression in NB cells and tissues. **A** Images of immunohistochemical staining for ATRX on tissue sections from SK-N-AS or SK-N-FI xenografts with or without ONC201 treatment. **B** Immunoblots show ATRX expression after ONC201 treatment in SK-N-AS, SK-N-FI, and SK-N-AS ρ^0^ cells. Data are presented as means ± SEM, and differences are considered significant at *P < 0.05. A two-group comparison was conducted utilizing an unpaired two-tailed t-test. **C** Representative profiles of ATRX immunostaining in human NB tissues. **D** Histological scoring of ATRX intensity in NB tissues. Dots represent the numbers in each group, and colors indicate different groups

ONC201 treatment previously was able to significantly reduce MYCN protein expression, inhibit tumor formation, and reactivate alpha thalassemia mental retardation X-linked (ATRX) expression in *MYCN*-amplified NB cell-derived xenograft tumors [5]. However, there have been no studies of ATRX expression in non-*MYCN*-amplified NB xenografts and cell lines. Our IHC analysis results showed that ONC201 did not reduce ATRX expression in xenograft tumors (Fig. 4A); in vitro experiments showed that ONC201 significantly reduced ATRX protein levels in SK-N-AS, SK-N-FI, and ρ^0^ cell groups (Fig. 4B). Furthermore, we evaluated ATRX protein expression and established IHC grading in resected tumors from NB patients. Despite the quantitative score of ATRX staining indicating a correlation with the maturity of neuroblastoma cells, it did not achieve a statistically significant result (Fig. 4C and D). Taken together, these observations showed that, regardless of its other effects, ONC201 substantially decreased ATRX expression in non-*MYCN*-amplified cell lines. However, further research is needed to determine whether the loss of ATRX is associated with epigenetic landscape reprogramming, as well as increased levels of telomerase reverse transcriptase (TERT) and alternative lengthening of telomeres (ALT) expression, in glioma subtypes [19, 20].

### ONC201 suppressed endothelial cell proliferation in vitro but did not repress tumor neovascularization in non-MYCN-amplified NB xenograft tumors

**Fig. 5:**
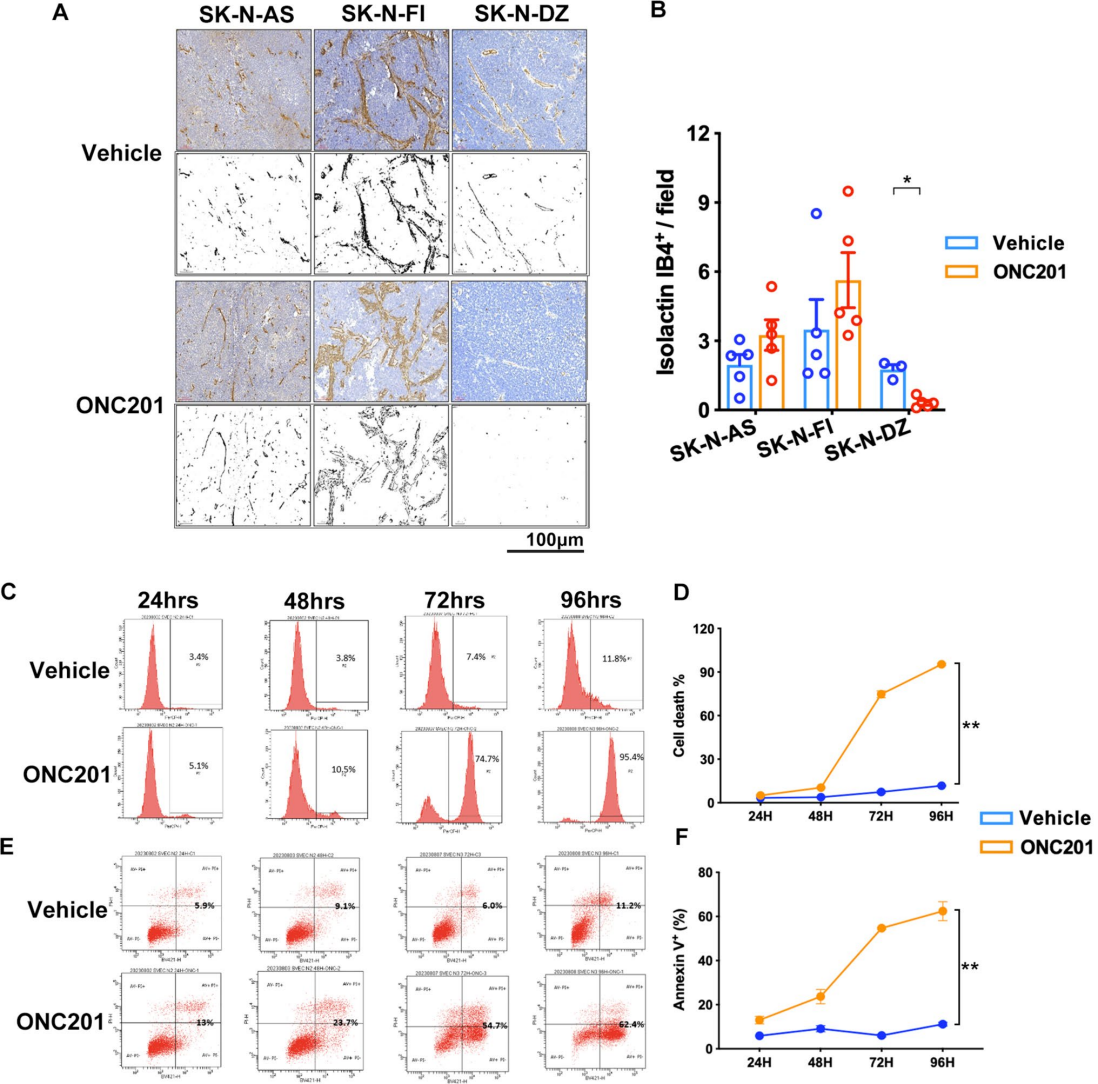
Effects of ONC201 in murine endothelial cells. **A** and **B** NB xenograft tissue sections were stained to identify IB4 and then counterstained with hematoxylin. IB4 staining areas were examined in ImageJ to quantify the percentage of endothelial coverage. Data are presented as means ± SEM, and differences are considered significant at *P < 0.05. A two-group comparison was conducted utilizing an unpaired two-tailed t-test. SVEC4 - 10 cells were treated with 15 µM ONC201 for 24–96 h, and flow cytometry was performed to determine the proportion of cell death via Trypan blue assay (**C** and **D**) and staining with annexin V (fluorescein isothiocyanate) and propidium iodide (PI) (**E** and **F**). Dots represent the numbers in each group, and colors indicate different groups. For (**D**) and (**F**), time course analysis is presented as mean ± SEM, considered significant at **P < 0.01, and calculated by ordinary two-way ANOVA and Tukey’s multiple comparison test

The pathological features of solid tumors, such as tumor expansion and progression, are dependent on vascularization [21]. Therefore, we examined whether ONC201 is related to tumor neoangiogenesis. The results of staining for the endothelial cell-specific marker isolectin IB4 showed that there were no significant differences in the number of blood vessels in non-*MYCN*-amplified NB cell lines or xenograft tumor tissue sections, regardless of treatment with ONC201, whereas ONC201 significantly reduced the number of blood vessels in the SK-N-DZ group (Fig. 5A and B). The effects of ONC201 on SVEC4 - 10 endothelial cells were assessed over time, showing a progressive increase in apoptosis (24–96 h). The trypan blue exclusion assay demonstrates a progressive increase in apoptosis following ONC201 treatment at 24, 48, 72, and 96 h (Fig. 5C and D). Flow cytometry using annexin V and PI staining further validated this, revealing an increase in early (annexin V-positive) and late-stage (annexin V/PI double-positive) apoptosis (Fig. 5E and F). These findings highlight the pro-apoptotic effect of ONC201, which intensifies over time.

## Discussion

The results of this study showed that ONC201 promoted the expression of c-Myc and LGR5 in human NB tissues but decreased ATRX expression in both non-*MYCN*-amplified SK-N-AS and SK-N-FI NB xenografts. Furthermore, ONC201 failed to decrease the number of tumor vessels in non-*MYCN*-amplified SK-N-AS and SK-N-FI xenografts, whereas it induced a significant decrease in neovascularization within *MYCN*-amplified SK-N-DZ xenografts. We also found increased LGR5 expression and decreased ATRX expression in SK-N-AS ρ^0^ cells after treatment with ONC201, similar to wild-type SK-N-AS cells. These results suggested that ONC201 has an extra-mitochondrial function.

The transcription factor c-Myc, which is constitutively expressed in over 70% of human cancers, plays a crucial role in global metabolic reprogramming associated with malignant transformation [22]. Myc protein expression is related to poor prognosis and indicates a more aggressive cancer phenotype in NB [23]. In NB, the c-Myc oncogene triggers focal enhancer amplification or genomic rearrangements, which results in enhancer hijacking and the transformation of NB precursor cells within a transgenic animal model Zimmerman [24]. Increased levels of c-Myc can activate multiple downstream genes, which enhances cell cycle progression involving DNA synthesis and leads to more chromosomal abnormalities. These processes collectively contribute to genomic instability and resistance to chemotherapy [25]. In the present study, ONC201 induced protein expression of c-Myc, both in vitro and in vivo. Purhonen et al. [26] reported that complex III deficiency triggers upregulation of c-Myc expression, followed by excessive anabolic metabolism and increased cell proliferation, under conditions of energy and biosynthetic precursor deficiency [26]. Our previous study showed that ONC201 downregulated the mitochondrial respiratory chain subunits SDHB and NDUFS1, resulting in energy deficiency and further trigger c-Myc expression [5], similar to the results reported by Purhonen et al. [26], although the precise mechanism remains unclear.

LGR5, a stem cell marker, is overexpressed in several types of cancer, including glioblastoma, cervical, breast, and colorectal cancer [27–30]. LGR5 enhances cell movement, tumor development, and the epithelial-mesenchymal transition in breast cancer cells [29]. It is found in elevated levels in high-grade NB [31, 32] and functions as a precursor to the MEK/ERK and Akt survival signaling pathways, which are frequently activated in primary NB tumors [33]. LGR5 protein promoted acceleration of the cell cycle; its expression was positively correlated with the expression levels of c-Myc, β-catenin, and cyclin D1 [18]. Our findings regarding simultaneous upregulation of c-Myc and LGR5 expression after ONC201 treatment suggested a causal relationship between the two oncogenic molecules. Further studies are required to determine whether LGR5 triggers c-Myc expression or vice versa.

ATRX has been implicated in neuronal differentiation and functions as a tumor suppressor, protecting cells from DNA replication stress by resolving difficult-to-replicate G-quadruplex DNA structures [34]. In our previous study, both ONC201 treatment and the genetic attenuation of ClpP and ClpX expression resulted in significant upregulation of the tumor suppressor ATRX, promoting neurite outgrowth in *MYCN*-amplified NB cells [5]. In the present study, we showed the association of ATRX expression with maturation of human NB tissues. However, ONC201 not only downregulated ATRX expression in non-*MYCN*-amplified NB cells in vitro, but also found that ONC201 did not achieve upregulate ATRX in non-*MYCN*-amplified xenografts, whereas it contributed to significant upregulation of ATRX in *MYCN*-amplified SK-N-DZ xenografts [5]; this upregulation may have been related to a decrease in MYCN protein expression. The failure of c-Myc downregulation after ONC201 treatment in this study was presumably responsible for failed ATRX upregulation in vitro and in vivo, although the precise mechanisms remain unknown.

Tumors undergo rapid neovascularization to support the rapid proliferation of cancer cells. In this study, we showed that ONC201 treatment failed to decrease tumor vessel formation in non-*MYCN*-amplified SK-N-AS and SK-N-FI xenografts, whereas it elicited a significant decrease in neovascularization among *MYCN*-amplified SK-N-DZ xenografts. Myc protein is recognized for its role in enhancing cell growth and transformation, in addition to its influence on vascular systems and blood formation, by acting as a primary controller of factors that stimulate angiogenesis [35]. Furthermore, knockdown of LGR5 by small interfering RNA (siRNA) can inhibit angiogenesis in gastric cancer [36]. In the present study, ONC201 treatment did not suppress either c-Myc or LGR5 expression, which may have led to the failed decrease in tumor vessel formation within non-*MYCN*-amplified NB xenografts.

In this study, we used EtBr to deplete mtDNA and generate ρ^0^ cells. The response of SK-N-AS ρ^0^ cells to ONC201 treatment was almost identical to that of SK-N-AS cells in terms of LGR5 upregulation and ATRX downregulation. Depletion of mtDNA induces prostate cancer progression through increased PI3 K/Akt2 signaling [37]. We suspect that LGR5 upregulation by ONC201 treatment follows a similar mechanism, involving mtDNA deregulation regardless of its depletion by EtBr. Indeed, complexome profiling of ρ^0^ cells identified 1002 mitochondrial proteins, revealing changes in the abundance and organization of numerous multiprotein complexes, such as previously undescribed assemblies [38].

Either LGR5 upregulation or ATRX downregulation would likely be affected, but this suspicion awaits further verification. The failure to increase c-Myc expression in SK-N-AS ρ^0^ cells, compared with parental SK-N-AS cells, may have been due to c-Myc stimulation of nuclear-encoded mitochondrial genes and mitochondrial biogenesis [39]. Mitochondrial depletion in NB cells disrupts feedback loops in a manner likely to be different from that in various NB subtype cells, and c-Myc will continue to increase in the latter cells in response to ONC201 treatment. However, further research is needed to verify this hypothesis.

## Conclusion

The results of the present study indicated that ONC201 exerts oncogenic, rather than tumor suppressive, effects in non-*MYCN*-amplified NB cells and xenografts. These effects involve continued tumor growth and the upregulation of c-Myc and LGR5 expression along with ATRX downregulation, as well as failure to decrease tumor neovascularization. We suspect that ONC201 has extra-mitochondrial effects in addition to its disruption of mitochondrial function in subsets of NB cells and tissues.

## Supplementary Information

Below is the link to the electronic supplementary material.

Supplementary file1 (PDF 1911 kb)

## Data Availability

Data from this study can be obtained from the corresponding author upon request. They are not publicly accessible due to privacy concerns.
