# Supplementary material for: ONC201 exerts oncogenic effects beyond its mitochondria-disturbing role in neuroblastoma subsets
Source: J Mol Med (Berl). 2025 Apr 10;103(5):571–82. doi: 10.1007/s00109-025-02541-0 (PMC12078449; doi:10.1007/s00109-025-02541-0)

(Fig.4) Supplement data
C-myc and Actin

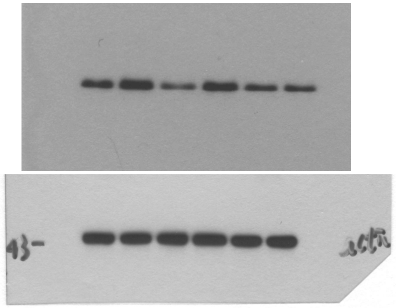

(Fig.5) Supplement data
ATRX and Actin

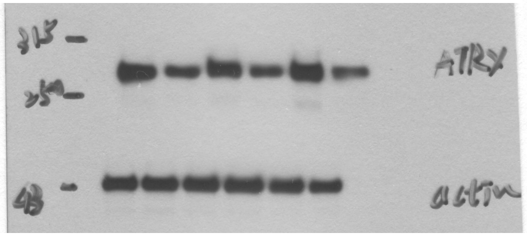

(Fig.4) Supplement data
LGR5 and Actin

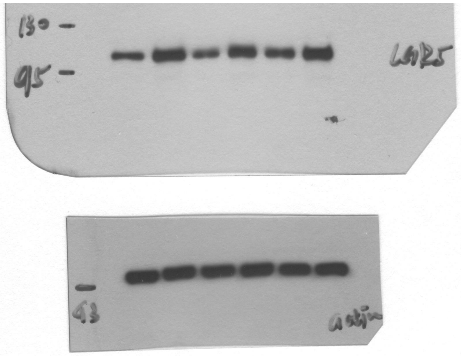

Supplement: Supplementary file 1 — Supplementary file1 (PDF 1911 kb) [file 109_2025_2541_MOESM1_ESM.pdf]
